# Validation of computational determination of microsatellite status using whole exome sequencing data from colorectal cancer patients

**DOI:** 10.1186/s12885-019-6227-7

**Published:** 2019-10-21

**Authors:** Amanda Frydendahl Boll Johansen, Christine Gaasdal Kassentoft, Michael Knudsen, Maria Bach Laursen, Anders Husted Madsen, Lene Hjerrild Iversen, Kåre Gotschalck Sunesen, Mads Heilskov Rasmussen, Claus Lindbjerg Andersen

**Affiliations:** 1 Department of Molecular Medicine, Aarhus University Hospital, Palle Juul-Jensens Boulevard 99, DK-8200 Aarhus N, Denmark; 2 Department of Surgery, Herning Regional Hospital, Herning, Denmark; 3 Department of Surgery, Aarhus University Hospital, Aarhus, Denmark; 4 Department of Surgery, Randers Regional Hospital, Randers, Denmark

**Keywords:** MSIsensor, Colorectal cancer, DNA mismatch repair deficiency, Microsatellite instability, MSI, MSS, POLE, Exome sequencing

## Abstract

**Background:**

Microsatellite instability (MSI), resulting from a defective mismatch repair system, occurs in approximately 15% of sporadic colorectal cancers (CRC). Since MSI is associated with a poor response to 5-fluorouracile based chemotherapy and is a positive predictive marker of immunotherapy, it is routine practice to evaluate the MSI status of resected tumors in CRC patients. MSIsensor is a novel computational tool for determining MSI status using Next Generation Sequencing. However, it is not widely used in the clinic and has not been independently validated in exome data from CRC. To facilitate clinical implementation of computational determination of MSI status, we compared MSIsensor to current gold standard methods for MSI testing.

**Methods:**

MSI status was determined for 130 CRC patients (UICC stage I-IV) using immunohistochemistry, PCR based microsatellite stability testing and by applying MSIsensor to exome sequenced tumors and paired germline DNA. Furthermore, we investigated correlation between MSI status, mutational load and mutational signatures.

**Results:**

Eighteen out of 130 (13.8%) patients were microsatellite instable. We found a 100% agreement between MSIsensor and gold standard methods for MSI testing. All MSI tumors were hypermutated. In addition, two microsatellite stable (MSS) tumors were hypermutated, which was explained by a dominant POLE signature and pathogenic POLE mutations (p.Pro286Arg and p.Ser459Phe).

**Conclusion:**

MSIsensor is a robust tool, which can be used to determine MSI status of tumor samples from exome sequenced CRC patients.

## Background

Colorectal cancer (CRC) is the third most common cancer worldwide and the second leading cause of cancer-related deaths [1]. The UICC Tumor-Node-Metastasis (TNM) staging is the general parameter used for guiding prognosis and treatment of CRC patients [2]. In addition, the molecular subtype of the tumor influences treatment decisions and outcome. While most sporadic CRC tumors develop through the chromosomal instable (CIN) pathway, close to 15% develop via the microsatellite instability (MSI) pathway [3, 4]. Moreover, MSI is a hallmark of hereditary Lynch-syndrome related cancer [5]. MSI is caused by a deficient mismatch repair (dMMR) system resulting in hypermutation due to slippage of the DNA polymerase during replication. This is most evident in microsatellites structures, which are defined as repeating sequences of 2–6 nucleotides occurring throughout the genome [4]. Generally, patients with MSI tumors have a better prognosis than stage-matched microsatellite stable and CIN tumors [4]. Furthermore, while MSI patients respond inferiorly to standard 5-fluorouracile (5-FU) based chemotherapy [6], MSI is a positive predictive marker of immunotherapy [7]. Therefore, it is recommended to screen all resected CRC tumors for dMMR to stratify treatment options [8].

Routine testing for dMMR is performed by immunohistochemically (IHC) quantification of the MMR proteins MLH1, MSH2, MSH6 and PMS2 [9–12]. This is often complemented by a polymerase chain reaction (PCR) based assessment of the stability of a five quasi-monomorphic mononucleotide repeats, referred to as pentaplex PCR [8, 13–15]. Both methods are laborious, time-consuming, limited to a small set of analytical targets and to some extent involves subjective interpretation. With the increasing use of Next Generation Sequencing (NGS) in cancer diagnostics, various computational tools have been developed aiming to determine the microsatellite status using an increased number of microsatellite regions [16–18]. These tools have the potential to determine MSI status directly from NGS data, without the need for additional biological testing. The most widely used tool – MSIsensor [16] – has shown promising results [17, 19, 20]. So far, the reported MSIsensor results have primarily been produced using sequencing data from smaller cancer gene panels [20–23]. Hence, there is an unmet need to examine the performance of MSIsensor on whole exome sequenced data. Here, we benchmarked the accuracy of MSIsensor against gold standard IHC and pentaplex PCR analyses in a cohort of 130 exome sequenced CRC patients. We aimed to justify the use of MSIsensor in the clinic as a replacement of the current pentaplex PCR and IHC practice.

## Methods

### Samples

Patients with UICC stage I-IV CRC were recruited between May 2014 and January 2017 at the Surgical Departments of Aarhus University Hospital, Randers Hospital and Herning Hospital. Tumor and matched germline DNA from buffy coat were collected at surgery. In total, 130 CRC patients (Table 1) who underwent molecular testing, including microsatellite stability evaluation, were included in this study. Four patients presented with synchronous tumors. From these, we randomly selected one tumor. We note that synchronous tumors in all cases were classified alike by gold standard methods (IHC and pentaplex PCR) and MSIsensor (data not shown).

**Table 1:** Patient characteristics and demographics

Patients, n	130
Samples, n	134^a^
Age at surgery, median (range)	67.8 (43–91)
Gender, n (%)	
Female	56 (43)
Male	74 (57)
Pathological UICC stage, n (%)	
I	6 (4.6)
II	41 (31.6)
III	81 (62.3)
IV	2 (1.5)
MSS/MSI status, n (%)	
MSI	18 (14)
MSS	112 (86)

^a^Four patients had synchronous cancers. One sample was chosen randomly from each patient

### Immunohistochemical and pentaplex PCR assessment of microsatellite status

IHC was performed as part of the routine diagnostic work-up and the results were extracted from patient hospital files. In brief, the presence or absence of nuclear expression of MLH1, MSH2, MSH6 and PMS2 was assessed in the tumor cells. Tumors were defined as mismatch repair proficient if all four proteins were expressed and mismatch repair deficient if any of the four proteins were not expressed.

Analysis of MSI status by PCR was performed at Department of Molecular Medicine (Aarhus University Hospital) using a panel of the five mononucleotide microsatellite loci; BAT-25, BAT-26, NR-21, NR-22 and NR-24 as previously described [14, 15] (Additional file 1: Table S1). Tumors were classified as MSI when three or more markers showed instability, i.e. changed pattern compared to a normal control sample. If less than three markers were unstable, the tumors were classified as MSS. A sample was classified as MSI if any of the methods scored the sample as dMMR or MSI. Otherwise, the sample was classified as MSS.

### Whole exome sequencing

Paired tumor derived from freshly frozen or formalin-fixed paraffin-embedded tissue and germline DNA from buffy coat were sequenced using paired-end (2 × 150 bp) whole exome sequencing with the MedExomePlusV1_hg19 panel (Roche, 72.28 Mb), as previously described [24]. Sequencing adapters were bioinformatically removed using TrimGalore [25]. The trimmed reads were mapped to the reference genome (GRCh37/hg19) using BWA MEM [26]. PCR duplicates were flagged using Picard MarkDuplicates [27], and the alignment was further processed using GATK IndelRealigner and BaseRecalibrator according to the GATK Best Practices (v3.7) [28].

#### MSIsensor

We applied MSIsensor (version 0.5) using default parameters to facilitate interpretation and translation to other laboratory facilities. MSIsensor identifies somatically mutated microsatellite loci in NGS data using a two-step process, which first involves scanning the reference genome for microsatellite sites. Sites are considered as microsatellites only if the sequence motif is at most five bases long and repeated at least three times. Microsatellite sites with less than 20 mapped reads in tumor or germline are not considered. The second part of the analysis uses a χ^2^ test to identify mutated microsatellites by comparing the distributions of homopolymer lengths in the tumor and normal samples at the sites identified in the first step. The resulting MSIsensor score is a value between 0 and 100 that corresponds to the percentage of mutated microsatellite loci. The tumors were classified as MSI if the score was greater than or equal to 3.5 and MSS if less than 3.5, which is the suggested cut-off for exome sequenced samples in the original MSIsensor publication [16].

#### Mutational load and mutational signatures

Somatic variants (SNVs and INDELs) were called using GATK MuTect2 [28]. Variants that did not pass all MuTect2 filters were further evaluated and retained if called as high-confidence by VarScan2 [29]. Tumor mutational burden was calculated as the total number of variants per targeted mega base (Mb). We used k-means clustering to differentiate hypermutated tumors from non-hypermutated tumors.

COSMIC mutational signatures (Version 2) were computed using deconstructSigs [30]. All samples had a mutational sum greater than 50, thereby fulfilling the recommended criterion for assessing the mutational signature [30].

#### POLE mutation status and classification

Variants were annotated using SnpEff (version 4.3.1) [31] and filtered for non-synonymous POLE mutations including two bases into introns on both sides of each exon. Variants with an allele frequency less than 10% were discarded. The remaining variants were inspected in Integrated Genomics Viewer (version 2.4.9) [32] and classified as “pathogenic”, “likely pathogenic”, “variant of uncertain significance”, “likely benign” and “benign” according to the American College of Medical Genetics and Genomics (ACMG) guidelines [33] using Ingenuity Variant Analysis (version 5.4.20190121) [34]. Furthermore, it was evaluated whether the variant was a common somatic variant, defined as seen somatic more than three independent times in the literature, as an extra layer to the classification.

## Results

### MSIsensor accurately classify MSI status in CRC patients

One-hundred thirty CRC patients were enrolled in this study. The microsatellite status was initially determined by gold standard methods IHC (*n* = 126) and pentaplex PCR (*n* = 118) (Additional file 1: Table S2). We found high agreement between the methods (Cohens Kappa 0.96). As described in Methods, samples were classified as MSI if tested positive by either of the gold standard methods. From this, 18 patients (13.8%) were classified as MSI.

**Fig. 1:**
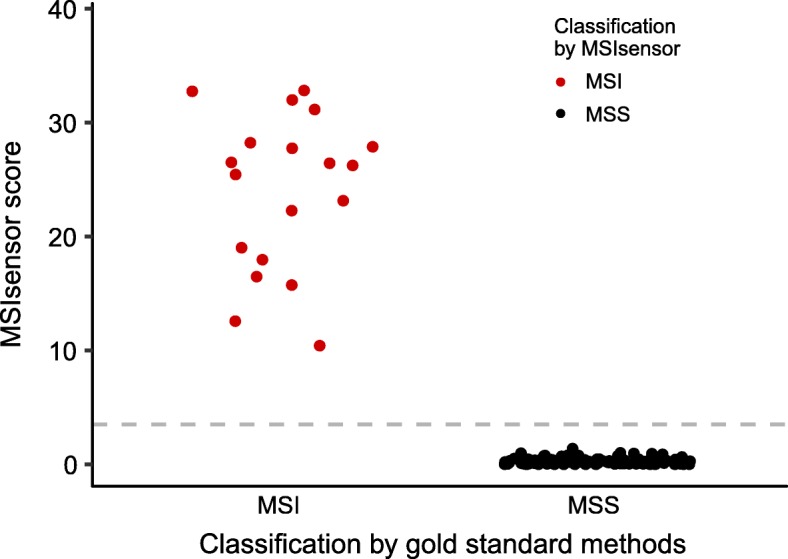
Distribution of MSIsensor scores. The distribution of MSIsensor scores according to classification by gold standard methods (pentaplex PCR and/or IHC). Red and black points indicate MSI and MSS tumors as classified by the MSIsensor, respectively. Dashed grey line shows the cut-off of 3.5% used to differentiate MSI from MSS

Using exome sequencing data from matched tumor and germline DNA from buffy coat, the MSIsensor scores were calculated and compared to microsatellite status determined by IHC and pentaplex PCR. With the recommended cut-off at 3.5, MSIsensor correctly classified all 130 patients into MSI (*n* = 18) and MSS (*n* = 112) (Fig. 1). The mean MSIsensor score was significantly different between MSI tumors (mean 24.2; range 10.4–38.6) and MSS tumors (mean 0.3; range 0–1.37) (*p* = 1.97 ∗ 10^− 10^, Welch Two Sample t-test).

### Sequencing duplicates influence the MSIsensor score

In the original publication by Niu et al., MSIsensor does not account for sequencing duplicates [16]. In order to investigate the effect of sequencing duplicates the flagged duplicates were removed prior to running the MSIsensor. The mean duplication rate for tumor and germline were 24.5% (range 10.2 -65.9%) and 11.2% (range 6.2 - 24.4%) respectively (Additional file 1: Table S3). If sequencing duplicates were not removed prior to application of MSIsensor, we observed an elevated MSIsensor score for 121 samples, a slight decrease for two samples while the MSIsensor score was unaltered for seven samples (Additional file 1: Table S3). The mean increase in MSIsensor score with sequencing duplicates were 2.65 (*p* = 6.46 ∗ 10^− 6^, paired t-test) for MSI samples and 0.3 (*p* = 6.57 ∗ 10^− 14^, paired t-test) for MSS samples. This translate to an 11% increase for MSI samples and 126% increase for MSS samples.

### MSIsensor classification is associated with hypermutation and dMMR mutational signatures

**Fig. 2:**
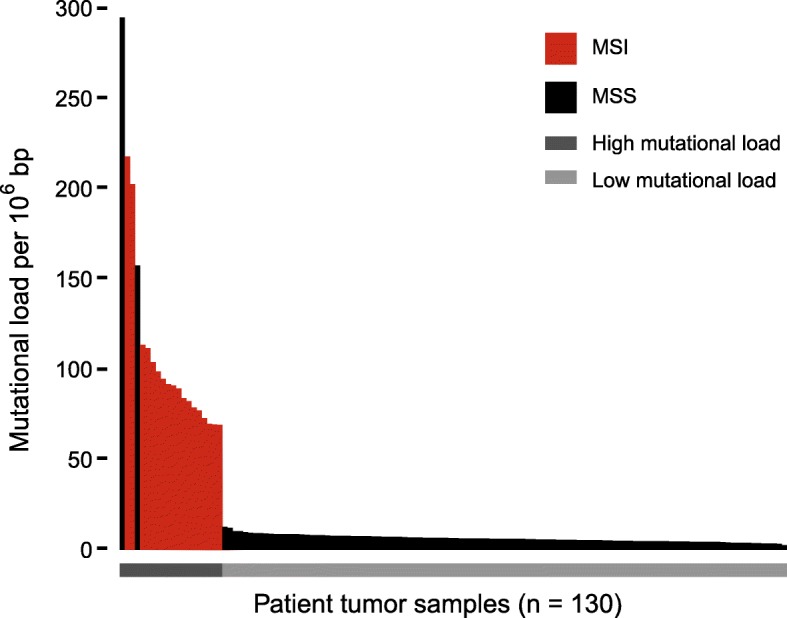
Mutational load of tumor samples. Mutational load per million bases (Mb) in tumor. Samples are ordered according to mutational load. Red bars indicate MSI tumors, whereas black bars indicate MSS tumors. Grey lines below the plot indicates the separation between hypermutated samples (dark grey) and samples with low mutational load (light grey)

**Fig. 3:**
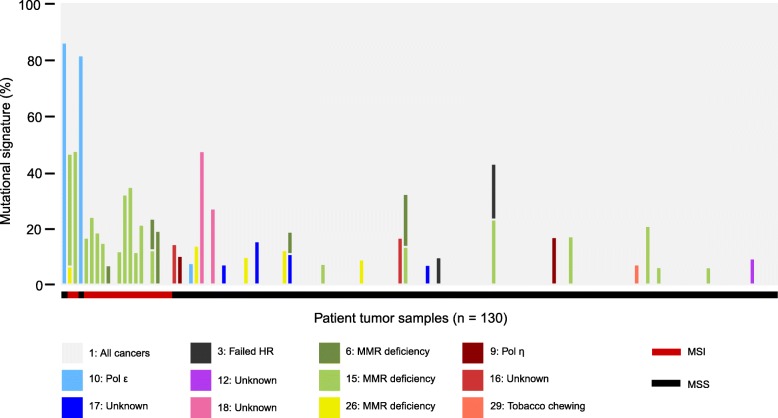
Mutational signatures of tumor samples. Cosmic mutational signatures of tumor samples, given in percentage (%). Samples are ordered according to mutational load (comparable to Fig. 2). Color of bar represent mutational signatures as shown in the legend with signature number and proposed etiology. The MSI status of the samples is denoted below the plot with red (MSI) or black (MSS) lines

MSI cancers are known to be hypermutated [4]. In agreement, we found significantly higher mutational load in MSI tumors classified by MSIsensor (median 90.1 mutations/Mb; range 69.2–217.8) as compared to MSS tumors (median 6.1 mutations/Mb; range 2.6–294.8) (*p* = 1.09 ∗ 10^− 11^, Wilcoxon rank sum test) (Fig. 2). We found significantly more dMMR-associated signatures (signatures 6, 15 and 26) in MSI (14 out of 18) as compared to MSS (12 out of 112) tumors (*p* = 8.96 ∗ 10^− 9^, Fishers Exact test) (Fig. 3, Additional file 1: Table S4). Interestingly, two MSS tumors had a hypermutation phenotype with more than 150 mutations/Mb (Patients 1 and 4). Mutational signature analysis of these tumors showed a dominant signature 10 (86 and 77.5%, respectively), which is characterized by an altered activity of polymerase ε (POLE) [35]. Mutational analysis of the exome data confirmed that both tumors had pathogenic POLE mutations (patient 1: p.Pro286Arg, patient 4: p.Ser459Phe, Additional file 1: Table S5) located in the exonuclease domain of POLE, which are known to cause a hypermutated phenotype [36, 37]. A third tumor (Patient 24) showed a minor contribution from POLE signature 10 (6.6%). However, the tumor was not classified as hypermutated (10.23 mutations/Mb, Fig. 2) and had no underlying somatic POLE mutation. We identified additional 12 tumors with potential pathogenic somatic POLE mutations (Additional file 1: Table S5). However, these mutations were all located outside the exonuclease domain and the tumors did not show any signs of a POLE signature.

## Discussion

Evaluation of MSI status is important for the assessment of prognosis [4] and response to standard 5-FU chemotherapy [6]. More recently, MSI testing has become important for the guidance of immunotherapy as FDA approved pembrolizumab for unresectable or metastatic MSI/dMMR tumors in 2017 [38].

In addition to MSI status, mutational load is also being investigated as a biomarker for immunotherapy [39–41]. Thus, MSI status as well as mutational load is likely to improve treatment stratification of cancer patients. The increasing use of NGS in the diagnostic work-up of cancer patients offers a great potential for assessing both MSI status as well as mutational load. Various tools have been developed to assess the MSI status based on NGS data [16–18]. Here, we aimed to provide sufficient evidence to use MSIsensor as the sole method for determination of MSI status, thereby offering an objective assessment of MSI status.

Currently, IHC and pentaplex PCR are the methods of choice to determine MSI status in the clinic. Although widely used, discrepancy is commonly reported between the methods [42–44]. This was exemplified in our data where one sample was classified as MSS with IHC but as MSI using the pentaplex assay. Such inconsistencies demonstrate that both methods are indeed required to evaluate MSI status robustly in patients, and emphasizes the need for a single unambiguous method.

The majority of studies applying MSIsensor have used data from a small cancer specific panel (MSK-IMPACT [45]). Since the MSIsensor score is influenced by the distribution of microsatellite loci within a panel, these studies used a panel specific score of 10% to classify samples as MSI [19, 20]. Only a limited number of studies have applied MSIsensor on exome data [17, 46, 47], despite the fact that this is a widely used panel in cancer diagnostics. A study by Kautto et al. used exome data from TCGA (colon adenocarcinoma/rectal adenocarcinoma (COAD/READ) and uterine corpus endometrioid cancer (UCEC) cohorts) [17] to investigate the performance of various computational tools for MSI testing, including MSIsensor. This is partly the same data, which originally was used to developed MSIsensor (UCEC cohort) [16]. The current study is the first to validate the performance of MSIsensor in an independent exome sequenced cohort. In addition, to encourage MSIsensor implementation in routine laboratories, we used default settings similar to the original MSIsensor publication, including a cutoff threshold of 3.5. Our results documented excellent agreement between the classification by MSIsensor and orthogonal methods, suggesting that MSIsensor analysis of exome sequenced tumors may replace gold standard methods to assess the MSI status of CRC patients. As MSIsensor was originally developed using UCEC exome data, our validation in an independent CRC cohort further suggests that MSIsensor may be used in various exome sequenced cancers with success. The fact that MSIsensor has been successfully applied in a pan-cancer setting on data from MSK-IMPACT [19] sequenced samples supports this notion. However, further independent validations specifically in exome data from various cancers is warranted.

We have investigated how sequencing duplicates influence the MSIsensor score. We observed a significantly higher MSIsensor score when duplicates were not removed. The effect of sequencing duplicates on the MSIsensor score is most easily explained by PCR errors during NGS library preparation and sequencing. Homopolymeric loci are especially vulnerable in this regard, thus increasing the chance of obtaining significantly different length distributions between tumor and germline samples. Even though the MSI classification in our cohort was not altered, we recommend that researchers remove duplicates prior to application of MSIsensor to avoid false positive MSI classification.

While we found an excellent agreement between MSIsensor and gold standard methods to detect dMMR, the COSMIC mutational signatures did not identify all samples with dMMR. The COSMIC mutational signatures aim to classify mutational patterns associated with environmental and biological processes. A deficient mismatch repair system has been associated with signatures 6, 15, 20 and 26 [35, 48]. Signature 20 was not seen in any of our samples, which probably reflects its low frequency in cancers, in general [35]. We found dMMR signatures in 14 of the 18 (78%) MSI samples, while 12 out of 112 (10.7%) MSS samples also revealed signatures associated with dMMR. This clearly shows that mutational signatures cannot be used as a standalone test for determining whether a patient has a defective mismatch repair system. Rather, mutational signatures may be helpful in order to explain the underlying biological processes in the tumor. This was true for the two hypermutated samples with signature 10 (POLE signature, Patient 1 and 4), which had pathogenic POLE mutations. This information might be used for guiding the patients into clinical trials. Currently, clinical trials are enrolling patients with mutations in genes, POLE and POLD1, to determine the effectiveness of immunotherapy in these patients (ClinicalTrials.gov Identifier: NCT03461952).

## Conclusion

Here, we have validated MSIsensor as a robust tool, which can be used to determine the MSI status of tumor samples from exome sequenced CRC patients with standard settings and the recommended cut-off. We found a 100% agreement between MSIsensor and orthogonal gold standard methods (IHC and pentaplex PCR) for MSI testing. Thus, MSIsensor provide a cost-efficient method to facilitate the analysis of CRC patients, which can be integrated in routinely genetic testing of patients.

## Supplementary information

**Additional file 1: Table S1.** Primers and probes for pentaplex PCR. **Table S2.** Microsatellite status determined by various methods. **Table S3.** MSIsensor output from all patients. **Table S4.** Mutational signatures and MSI classification of patients. **Table S5.** POLE mutations identified in patient cohort.

## Data Availability

The datasets generated and/or analyzed during the current study are not publicly available due to Danish personal data protection regulations, but may be made available for specific analysis upon approval from the relevant Danish authorities.

## Abbreviations

5-FU: 5-fluorouracile
ACMG guidelines: American College of Medical Genetics
CIN: Chromosomal instable
COAD/READ: Colon adenocarcinoma/rectal adenocarcinoma
COSMIC: Catalogue of somatic mutations in cancer
CRC: Colorectal cancer
dMMR: Deficient mismatch repair
FDA: Food and Drug Administration
IHC: Immunohistochemistry
Mb: Mega base
MSI: Microsatellite instable
MSK-IMPACT: Memorial Sloan Kettering-Integrated Mutation Profiling of Actionable Cancer Targets
MSS: Microsatellite stable
NGS: Next Generation Sequencing
PCR: Polymerase chain reaction
POLD1: Polymerase delta 1
POLE: Polymerase epsilon
UCEC: Uterine corpus endometrioid cancer
